# Design of a Textile-Integrated Piezoelectric Braided Cord Sensor for Sleep Apnea Monitoring

**DOI:** 10.3390/mi17101200

**Published:** 2026-10-17

**Authors:** Yoshiro Tajitsu, Osamu Takeuchi, Saki Shimada, Takuto Nonomura

**Affiliations:** 1 Center for Innovation & Creativity, Kansai University, Osaka 564-8680, Japan; 2 Faculty of Foreign Language Studies, Kansai University, Osaka 564-8680, Japan; 3 Japan Research Laboratory of Sleep Science, Nishikawa Co., Ltd., Chuo, Tokyo 103-0006, Japan; shimada-saki@nishikawa1566.com (S.S.);

**Keywords:** poly-L-lactic acid (PLLA), piezoelectric braided cord, sleep apnea syndrome (SAS)

## Abstract

Piezoelectric braided cords composed of poly-L-lactic acid (PLLA) fibers can be integrated into textiles to detect mechanical signals without requiring sensors to be attached directly to the body. In this study, we investigated the potential of a bed-sheet-integrated piezoelectric braided cord sensor for unobtrusive physiological monitoring during sleep, using sleep apnea as an example of its possible applications. Finite element method (FEM) analysis was performed to qualitatively examine the directional characteristics of the piezoelectric response. Guided by these qualitative findings, a bed-sheet sensor with a multi-orientation stitching configuration was developed through iterative prototyping and experimental evaluation. Piezoelectric braided cord signals were recorded from 23 participants during their routine clinical polysomnography (PSG) examinations. The developed sensor enabled the detection of respiration- and pulse-related mechanical signals during sleep. During the initial analysis of data from a small number of these participants, we observed that a pulse-related periodic component remained detectable in the respiratory signal when respiratory motion decreased during apnea. Based on this observation, an apnea-related signal detection algorithm was developed and subsequently applied to the complete dataset of 23 participants. The sensor-based analysis was performed independently of the physicians’ PSG-based assessments. For the 18 participants with available physician-based apnea event assessments, the sensor-detected event counts were compared with PSG-based counts within 5-min intervals, without event-by-event temporal matching. These findings demonstrate the potential of the proposed sensing approach and provide a basis for future clinical investigations.

## 1. Introduction

Flexible sensors that can be integrated into textiles offer opportunities for detecting mechanical signals associated with human movement without attaching sensing devices directly to the body [1,2,3]. Piezoelectric braided cords composed of poly-L-lactic acid (PLLA) fibers are particularly suitable for such applications because they are flexible and can be stitched into fabrics [4,5]. In our previous studies, we have reported that the stitching configuration influences the mechanical deformation of the piezoelectric braided cord and its piezoelectric response [4,5].

**Figure 1:**
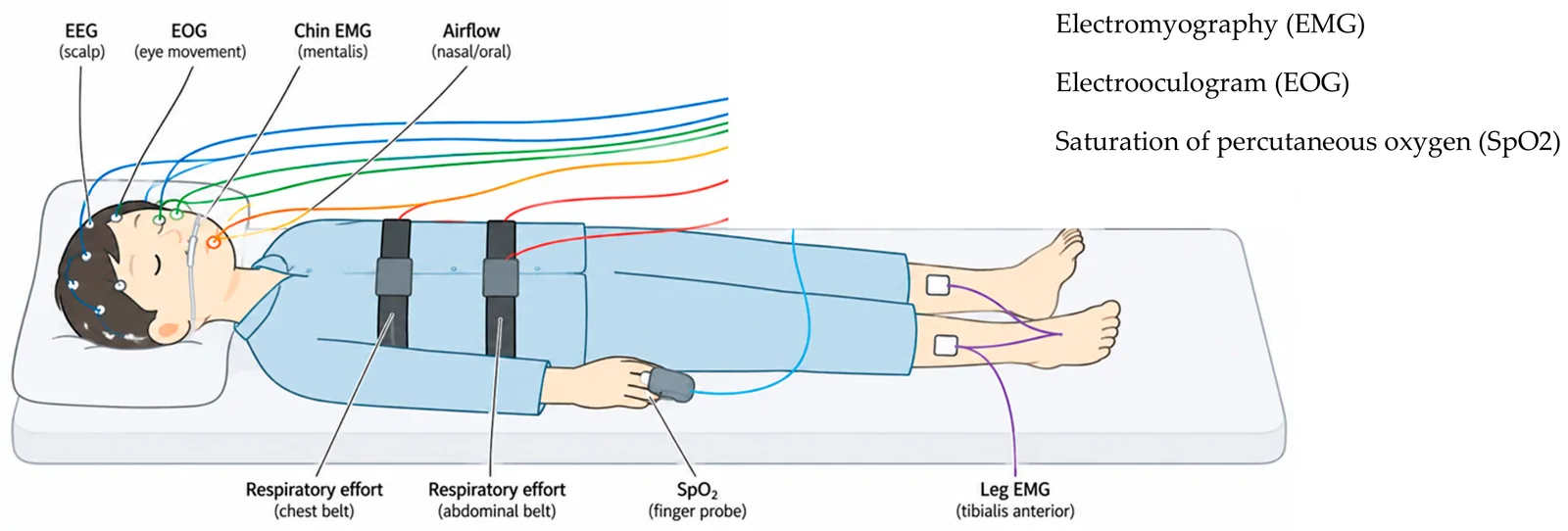
Polysomnography (PSG) setup.

One possible application of textile-integrated sensing is physiological monitoring during sleep. Respiration, cardiovascular pulsation, and body movements produce mechanical deformations that can be transmitted through bedding materials. Detecting these signals without direct sensor attachment may provide a useful approach to unobtrusive monitoring. Sleep apnea syndrome (SAS), characterized by repeated episodes of cessation or reduction in breathing during sleep, is an example of a condition in which such monitoring may be useful. SAS is associated with daytime sleepiness, an increased risk of accidents, hypertension, and cardiovascular diseases [6,7,8,9,10,11,12]. Obstructive sleep apnea (OSA) is a widespread sleep-related breathing disorder. A global epidemiological analysis estimated that approximately 936 million adults aged 30–69 years have mild to severe OSA, including approximately 425 million with moderate to severe OSA [13]. Polysomnography (PSG) is widely used for clinical evaluation of sleep apnea but generally requires an overnight examination at a medical facility, placing a burden on participants [14,15,16]. A typical PSG setup is illustrated in Figure 1. Textile-integrated sensors may contribute to future home-based monitoring of sleep apnea, and exploring this possibility is one of the motivations for the present study.

In the present study, finite element method (FEM) analysis was used to obtain qualitative guidance for sensor development. Based on the FEM findings and through iterative prototyping and experimental evaluation, a bed-sheet sensor incorporating piezoelectric braided cords stitched in multiple orientations was developed for sleep apnea monitoring.

During routine clinical PSG examinations involving 23 participants, signals from the piezoelectric braided cord sensor were recorded simultaneously with PSG, while the clinical examinations and assessments proceeded as usual. Respiration- and pulse-related signals were identified in the recorded sensor data, and their characteristics were used to develop an algorithm for detecting apnea-related signal changes. For 18 of the 23 participants, physician-based PSG apnea event assessments obtained from routine clinical practice were subsequently compared with the sensor-detected event counts within 5-min intervals. The findings suggested the potential usefulness of the developed sensor for sleep apnea monitoring and provided a basis for further investigation.

## 2. Piezoelectric PLLA Braided Cord Sensor

**Figure 2:**
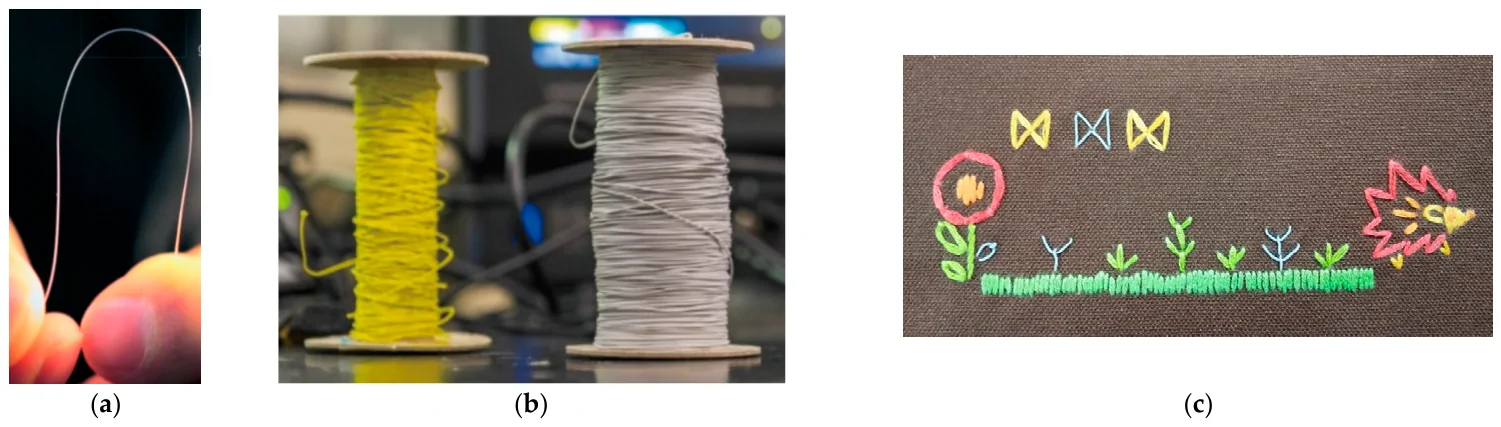
Structure of the piezoelectric PLLA braided cord sensor: (**a**) PLLA fiber; (**b**) piezoelectric PLLA braided cord; (**c**) stitched structure.

**Figure 3:**
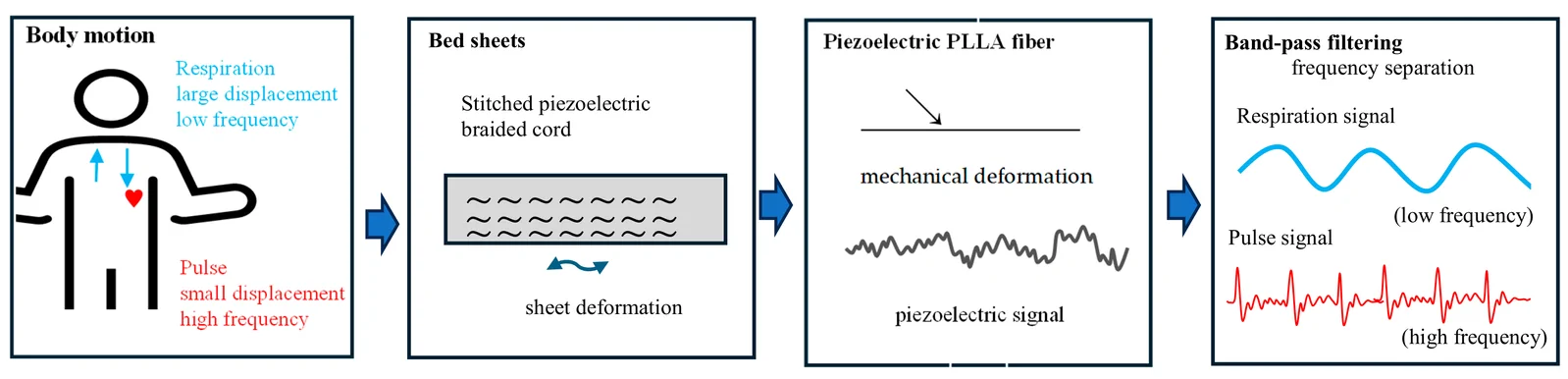
Sensing mechanism of the stitched piezoelectric PLLA braided cord sensor.

The sensor used in this study is based on a piezoelectric braided cord composed of PLLA fibers. PLLA is known to exhibit piezoelectricity when the polymer chains are strongly oriented along the fiber axis. In highly oriented PLLA fibers, mechanical deformation can generate electrical signals through the shear piezoelectric effect [17,18,19]. This property allows the fiber to function as a flexible piezoelectric sensing element without requiring electrical poling treatment [20,21,22,23,24,25,26]. To improve mechanical flexibility and durability, the PLLA fibers are arranged in a braided cord structure. The braided structure enables the sensor to maintain mechanical robustness while allowing sufficient deformation of the fibers when external forces are applied. The braided configuration provides mechanical stability and compatibility with textile-based applications, as shown in Figure 2. In this study, the piezoelectric PLLA braided cord is integrated into a bed sheet by stitching it onto the fabric using conductive yarn. This configuration allows the sensor to detect mechanical deformation of the sheet surface caused by body motion during sleep. When a person lying on the bed breathes or experiences cardiovascular pulsation, small mechanical displacements occur in the bed sheet, and these deformations are transmitted to the stitched braided cord structure. Because the braided cord is mechanically coupled to the sheet surface, deformation of the sheet induces mechanical deformation of the PLLA fibers inside the braided cord. Due to the piezoelectric effect of the oriented PLLA fibers, this mechanical deformation generates an electrical signal. The generated signal reflects the mechanical vibrations associated with physiological motions during sleep. Respiration typically produces relatively large displacements with low-frequency characteristics, whereas pulse signals appear as smaller displacements with higher-frequency components. Therefore, the electrical signal generated by the piezoelectric braided cord contains mixed physiological information, including respiration and pulse signals. These signals can be separated using band-pass filtering based on their frequency characteristics, as conceptually illustrated in Figure 3.

**Figure 4:**
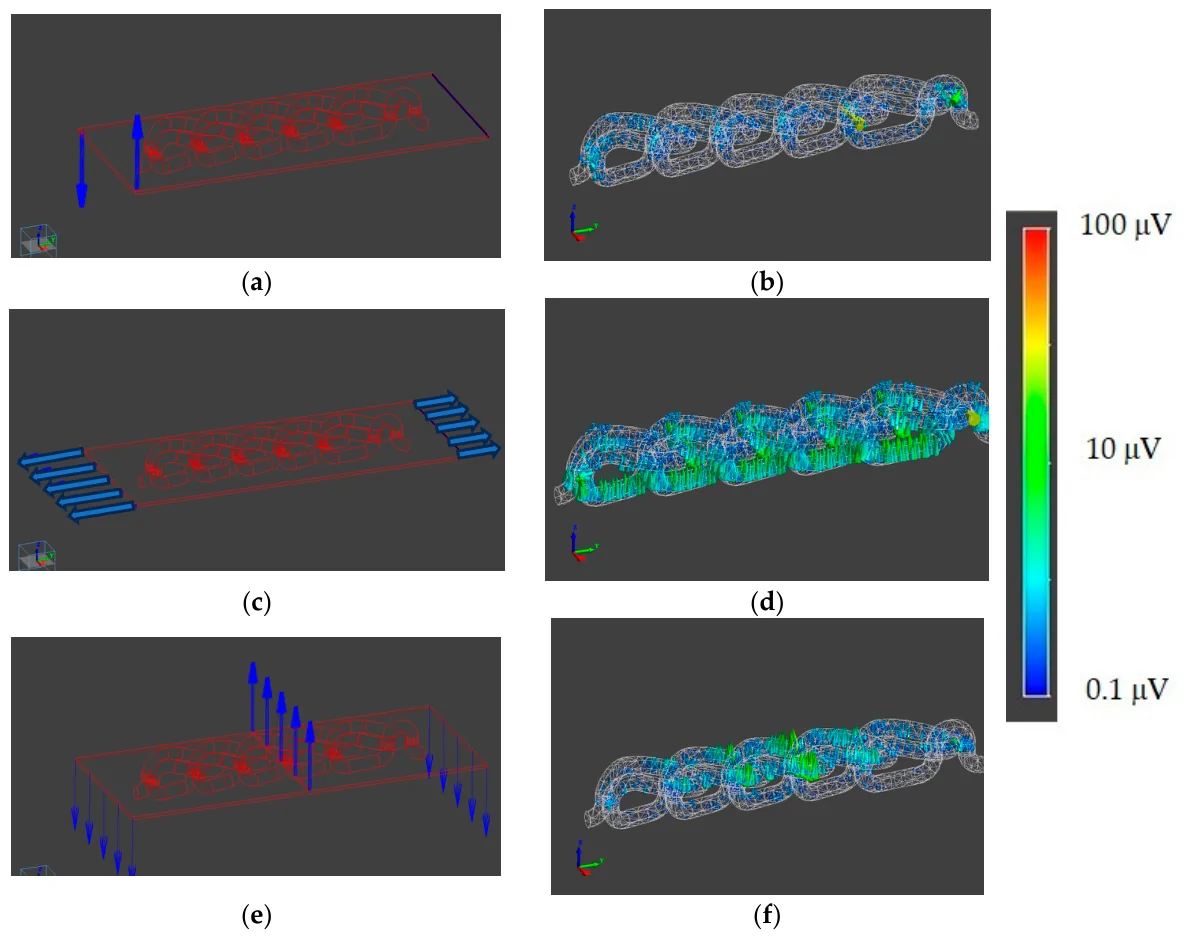
Piezoelectric responses of the stitch-patterned FEM model under different displacement conditions. (**a**,**b**) Displacement applied to an area outside the stitch and the corresponding piezoelectric response. (**c**,**d**) Uniform tensile displacement applied to the entire stitch and the corresponding piezoelectric response. (**e**,**f**) Localized displacement applied to the stitch and the corresponding piezoelectric response. The arrows indicate the directions of the applied displacement in the FEM analysis.

The FEM model and analytical procedure were based on our previously reported method [27,28], with modifications to the stitch configuration and applied displacement conditions for the present study. The FEM analysis was intended to qualitatively examine the effects of stitch geometry and displacement direction on the piezoelectric response, rather than to quantitatively predict the sensor output under actual sleeping conditions. The FEM analysis was performed using Femtet (version 10.0, Murata Software Co., Ltd., Yokohama, Japan). The theoretical basis of the finite element method is described in [29]. In the FEM model, the PLLA braided cord was treated as a homogeneous piezoelectric material using material properties based on previously reported values. The stitch locations were fixed, and mechanical displacement was applied to the model to examine the resulting piezoelectric response. The magnitude of the applied displacement was approximately 1% of the cord length. The stitch patterns were designed with reference to conventional embroidery structures [30,31]. Figure 4 shows the applied displacement conditions and the corresponding piezoelectric responses.

- When displacement was applied to an area where no stitch was present, almost no piezoelectric response was observed in the stitch.
- When tensile displacement was applied uniformly to the entire stitch, a relatively uniform piezoelectric response was observed along the stitch.
- When opposite displacements were applied locally to the stitch, piezoelectric responses with opposite polarities were observed.

**Figure 5:**
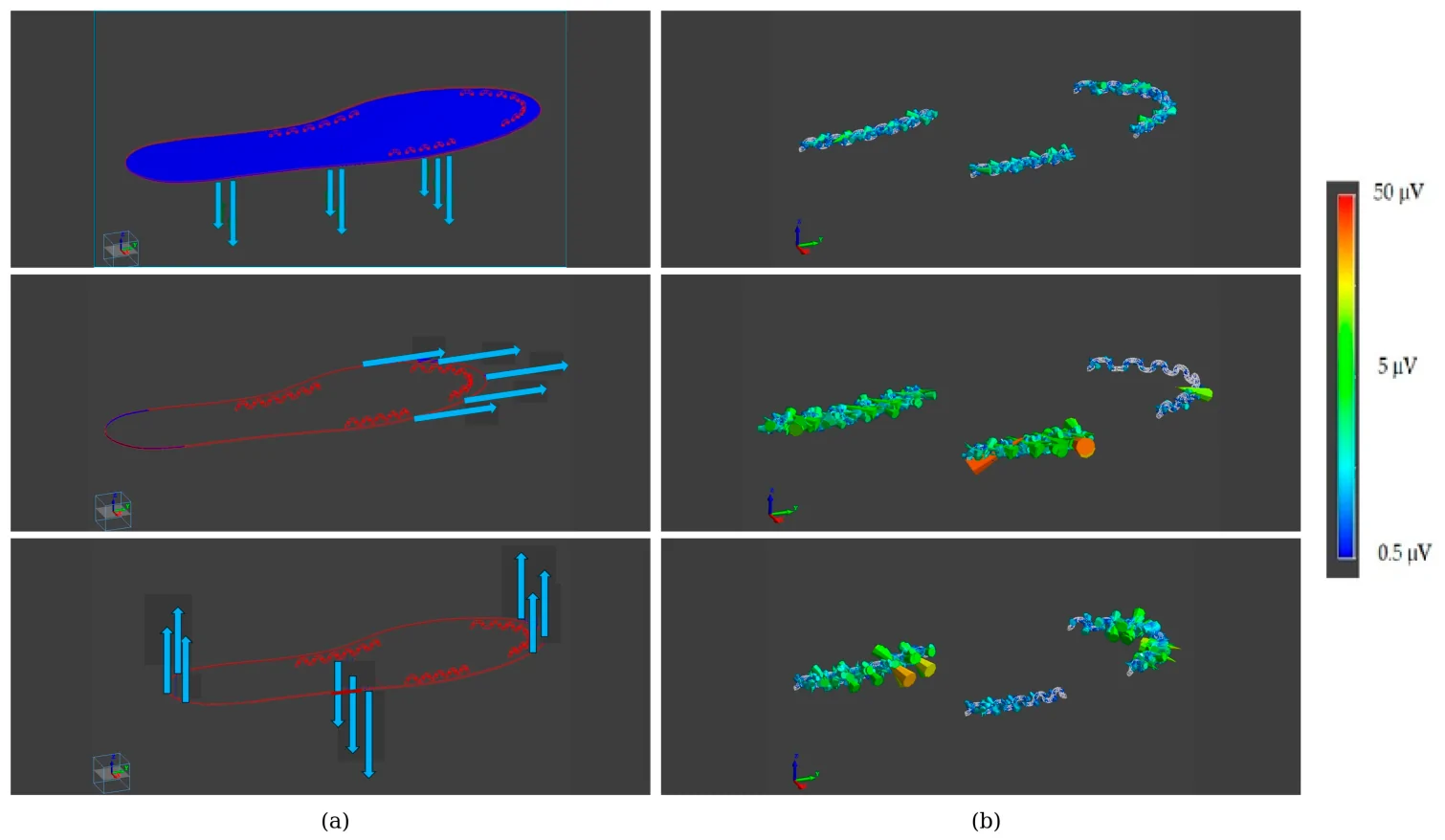
Effect of stitch geometry and displacement direction on the piezoelectric response. (**a**) FEM models with different stitch configurations and applied displacement conditions; (**b**) corresponding piezoelectric responses. The arrows indicate the directions of the applied displacement in the FEM analysis.

These FEM results demonstrate that the piezoelectric response of the stitched sensor depends on how and where the deformation is applied to the stitch. In practical textile applications, however, the stitched piezoelectric braided cord may have different orientations and may form curved as well as straight patterns. We therefore further investigated the effects of stitch orientation and geometry on the piezoelectric response. The results are shown in Figure 5. The FEM results showed that the piezoelectric response varied depending on the combination of stitch orientation and displacement direction. During sleep, the direction of deformation applied to the bed sheet varies with changes in body position and sleeping posture. Therefore, to reduce the dependence of the sensor response on the direction of deformation, the piezoelectric braided cord was stitched in multiple orientations. Based on this design concept, the sheet sensor used in this study was fabricated. These FEM results provide qualitative guidance for the selection of stitch configurations but do not establish the quantitative sensor response under actual sleeping conditions.

## 3. Prototype Sensor System

### 3.1. Bed Sheet Sensor

**Figure 6:**
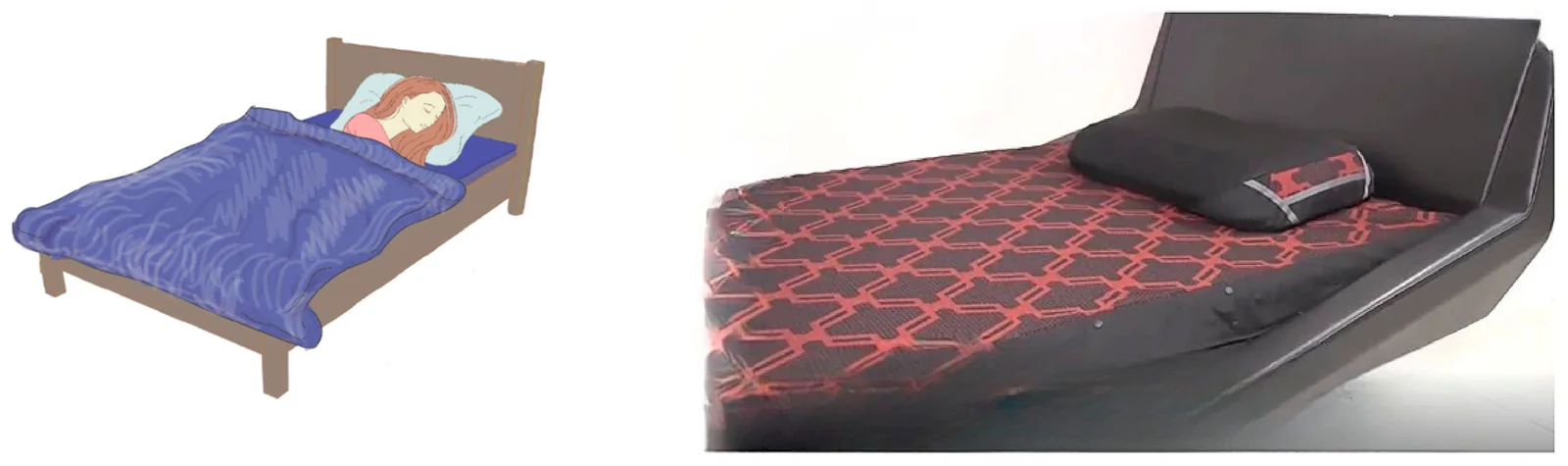
Prototype bed sheet sensor incorporating the piezoelectric PLLA braided cord used during PSG examinations.

Based on the design concept derived from the FEM analysis described above, the piezoelectric PLLA braided cord was stitched onto a bed sheet using an open stitch pattern combining chain and fly stitches. This stitch pattern incorporates multiple orientations of the braided cord to reduce the directional dependence of the sensor response. The final stitching configuration was determined through iterative prototyping and experimental evaluation, using the FEM results as qualitative design guidance. The prototype bed sheet sensor used during the PSG examinations is shown in Figure 6.

### 3.2. Circuit System for the Piezoelectric PLLA Braided Cord Bed Sheet Sensor

**Figure 7:**
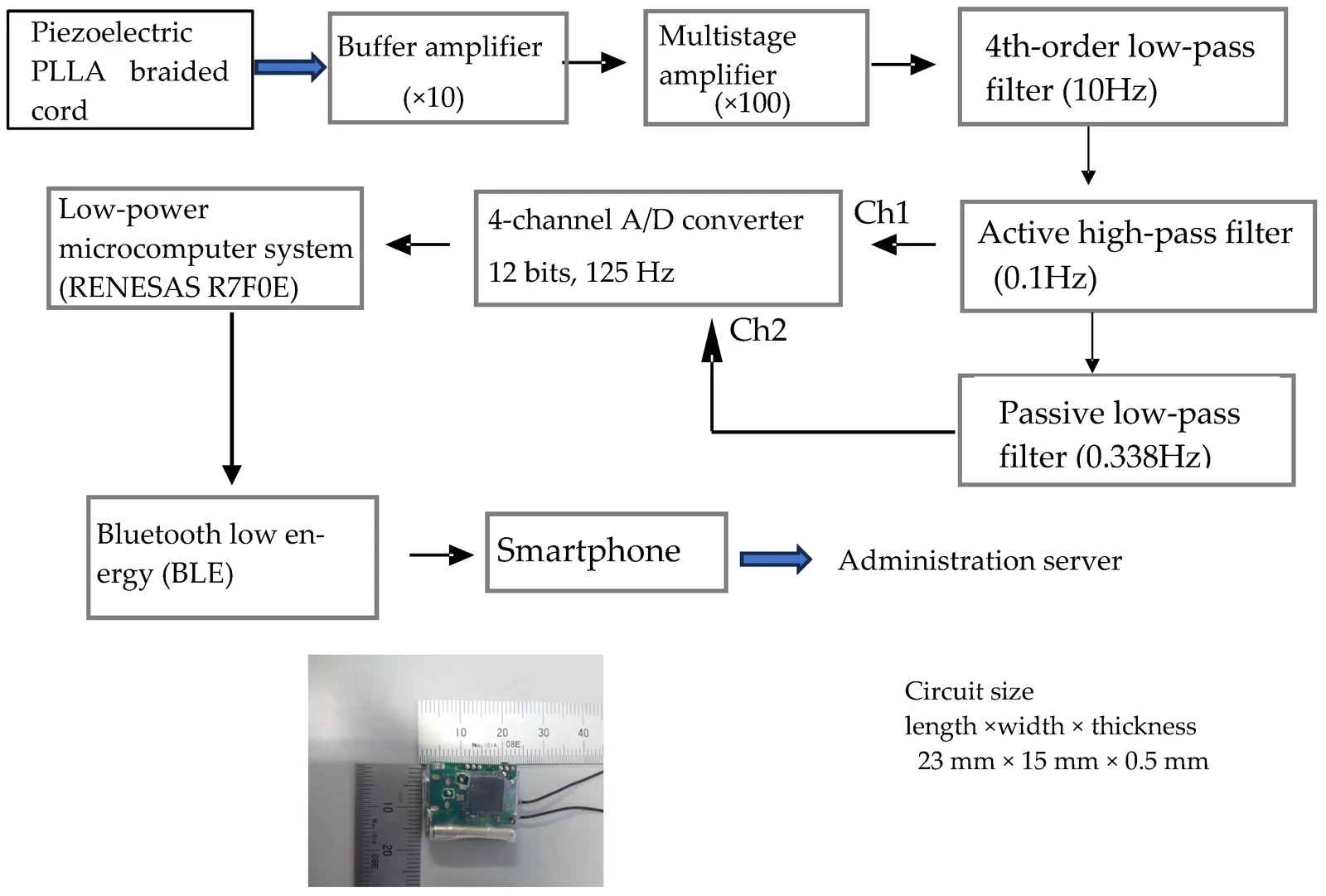
Circuit system for the piezoelectric PLLA braided cord bed sheet sensor.

Figure 7 shows the detection circuit and data transmission system developed for the bed sheet sensor. The battery-powered circuit consists mainly of amplification and filtering stages, an A/D converter, a low-power microcomputer, and a Bluetooth Low Energy (BLE) communication module. The circuit measures 23 mm × 15 mm × 0.5 mm. The acquired data are transmitted via BLE to a smartphone and subsequently transferred to a management server. Because the measurements were performed during routine PSG examinations at a sleep clinic, a data logger was also employed as a backup recording system to reduce the risk of data loss.

### 3.3. Signal Processing

The electrical signal generated by the piezoelectric braided cord contains both respiration- and pulse-related components, as described in Section 2. The signal acquired by the measurement circuit was digitized and processed using digital band-pass filtering to separate these physiological components based on their frequency characteristics. The respiration component was extracted using a band-pass filter in the range of 0.08–0.3 Hz, whereas the pulse component was extracted using a band-pass filter in the range of 0.9–8 Hz. The resulting filtered signals were used as the respiration and pulse waveforms, respectively. The overall sensing and signal separation concept is illustrated in Figure 3. For the apnea detection algorithm described in Section 4.2, the output of the analog low-pass filter with a cutoff frequency of 0.338 Hz was used directly to evaluate the reduction in respiratory signal amplitude and the remaining periodic component. The pulse frequency used for comparison was obtained separately from the pulse signal extracted using the 0.9–8 Hz band-pass filter.

## 4. Clinical Evaluation of the Proposed Sensor

During routine overnight PSG examinations at a sleep clinic, signals were recorded simultaneously using the bed-sheet-integrated piezoelectric braided cord sensor. Participants who provided informed consent underwent standard PSG while sleeping on a bed covered with the sheet incorporating the piezoelectric PLLA braided cord sensor. The study included 23 participants (18 males and 5 females) aged 15–81 years, as summarized in Table 1. The same bed-sheet sensor configuration was used for all 23 participants without modification during the clinical measurements. The clinical status of the participants was not disclosed to us during the measurements. The physician’s assessment of SAS was based on the apnea–hypopnea index (AHI) [32], as summarized in Table 2. Physician-based PSG apnea event assessments were available for 18 of the 23 participants and were used for the event-count comparison described in Section 4.3.

### 4.1. Signals from the Piezoelectric PLLA Braided Cord Sensor During Overnight Measurement

**Figure 8:**
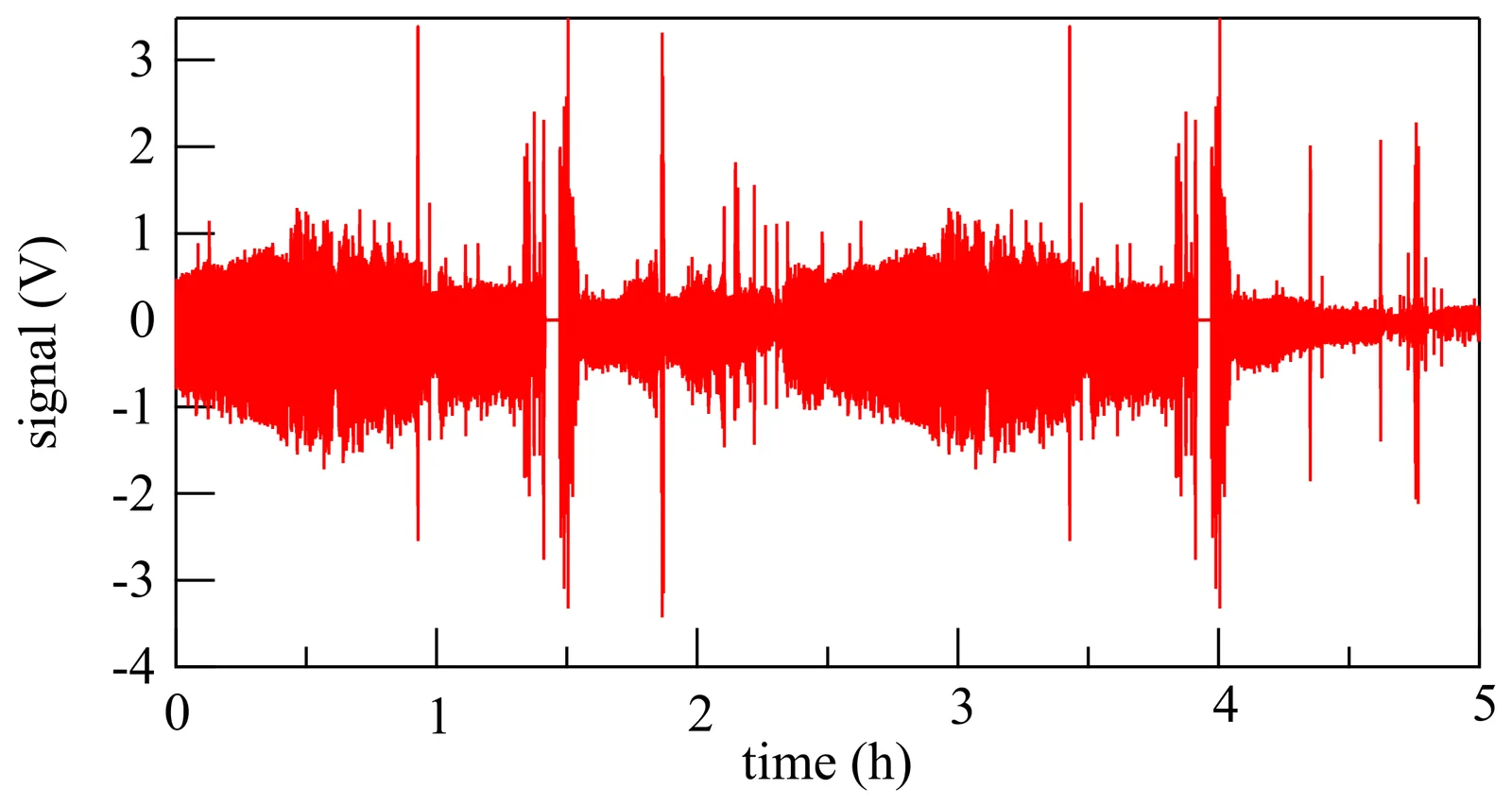
Representative signal recorded from the piezoelectric PLLA braided cord sensor during an overnight clinical measurement (19 September 2024, 0:00–5:00).

**Figure 9:**
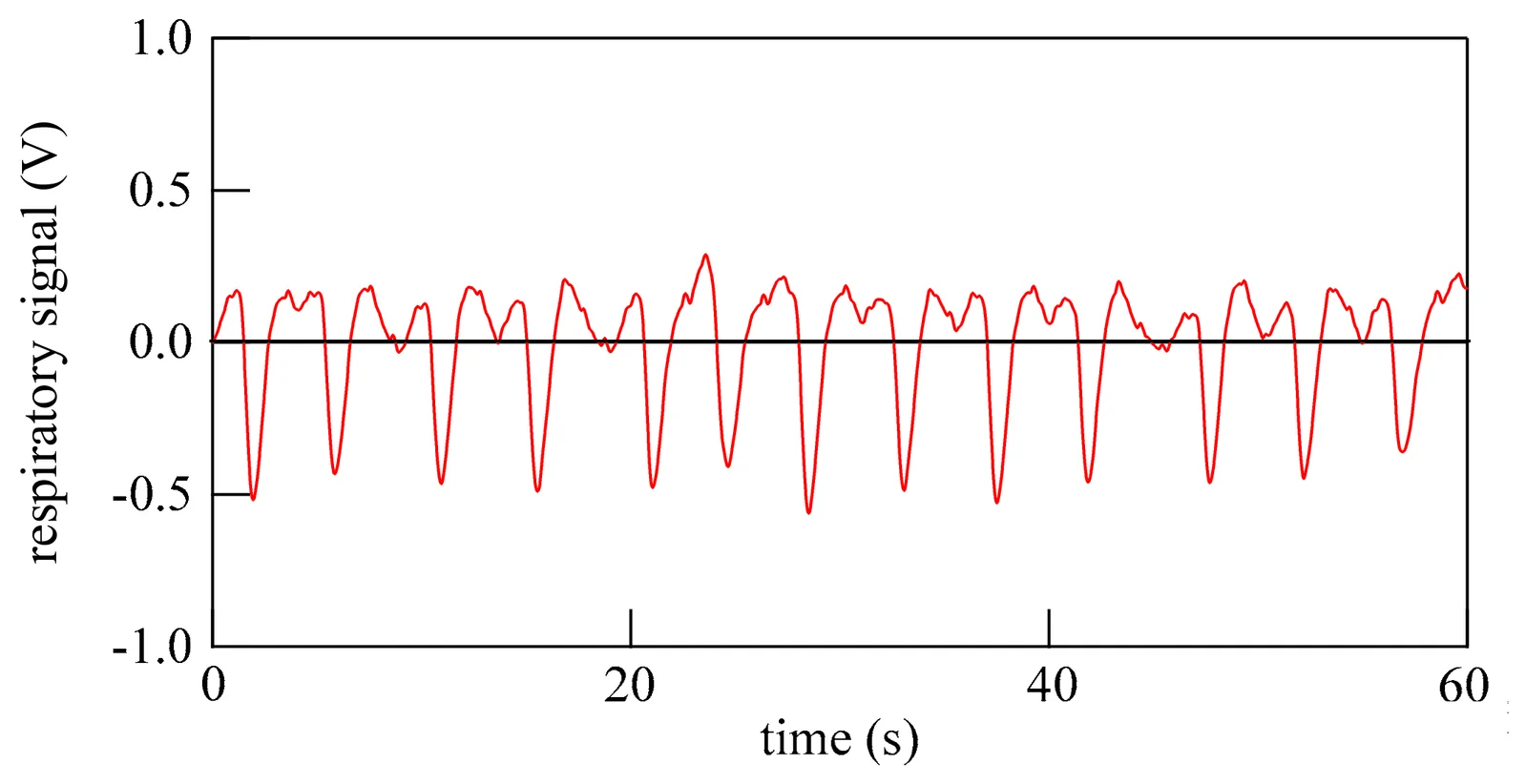
Sample 60-s of respiratory signals obtained from piezoelectric PLLA braided cord sensor.

**Figure 10:**
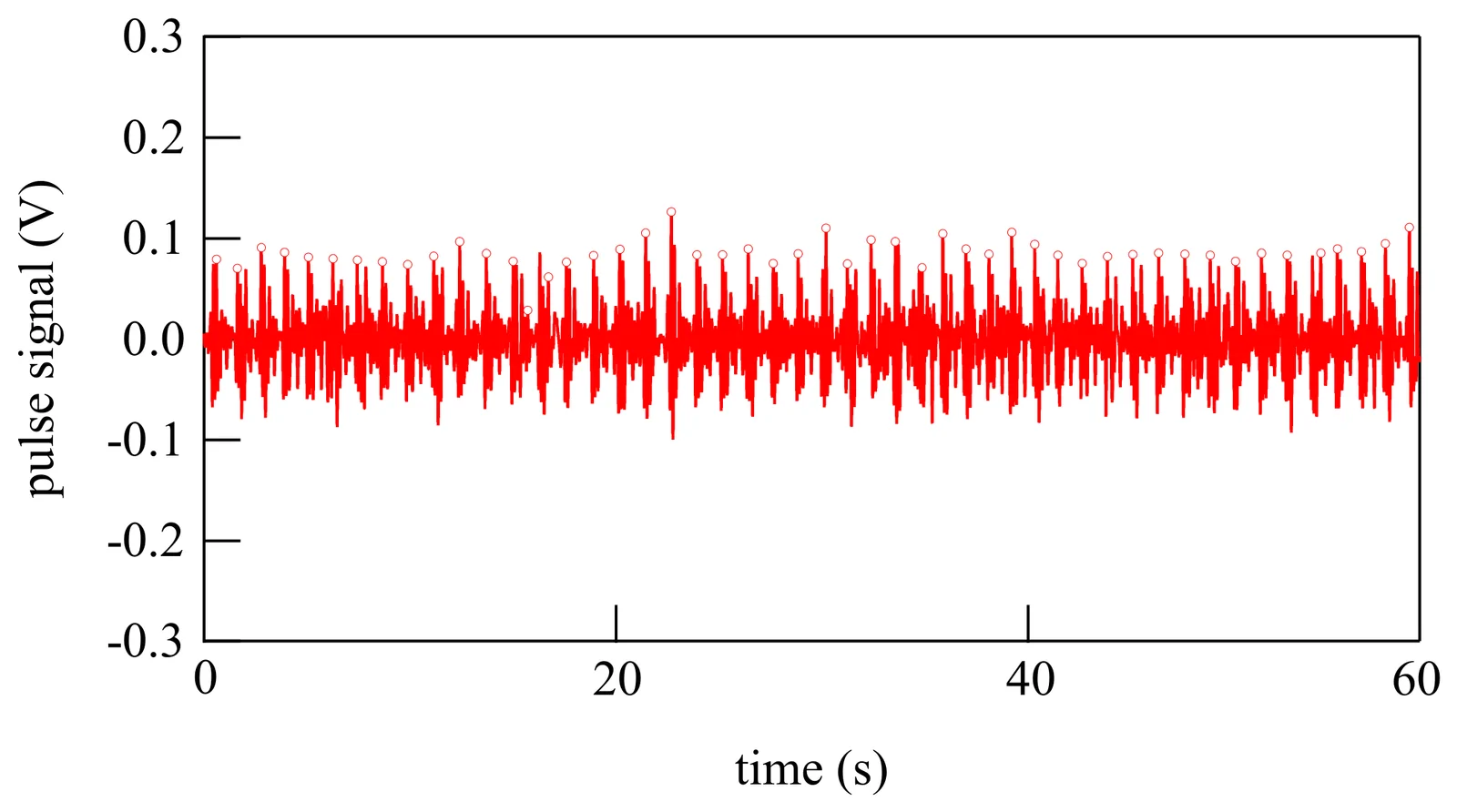
Sample 60-s of pulse signals obtained from piezoelectric PLLA braided cord sensor.

**Figure 11:**
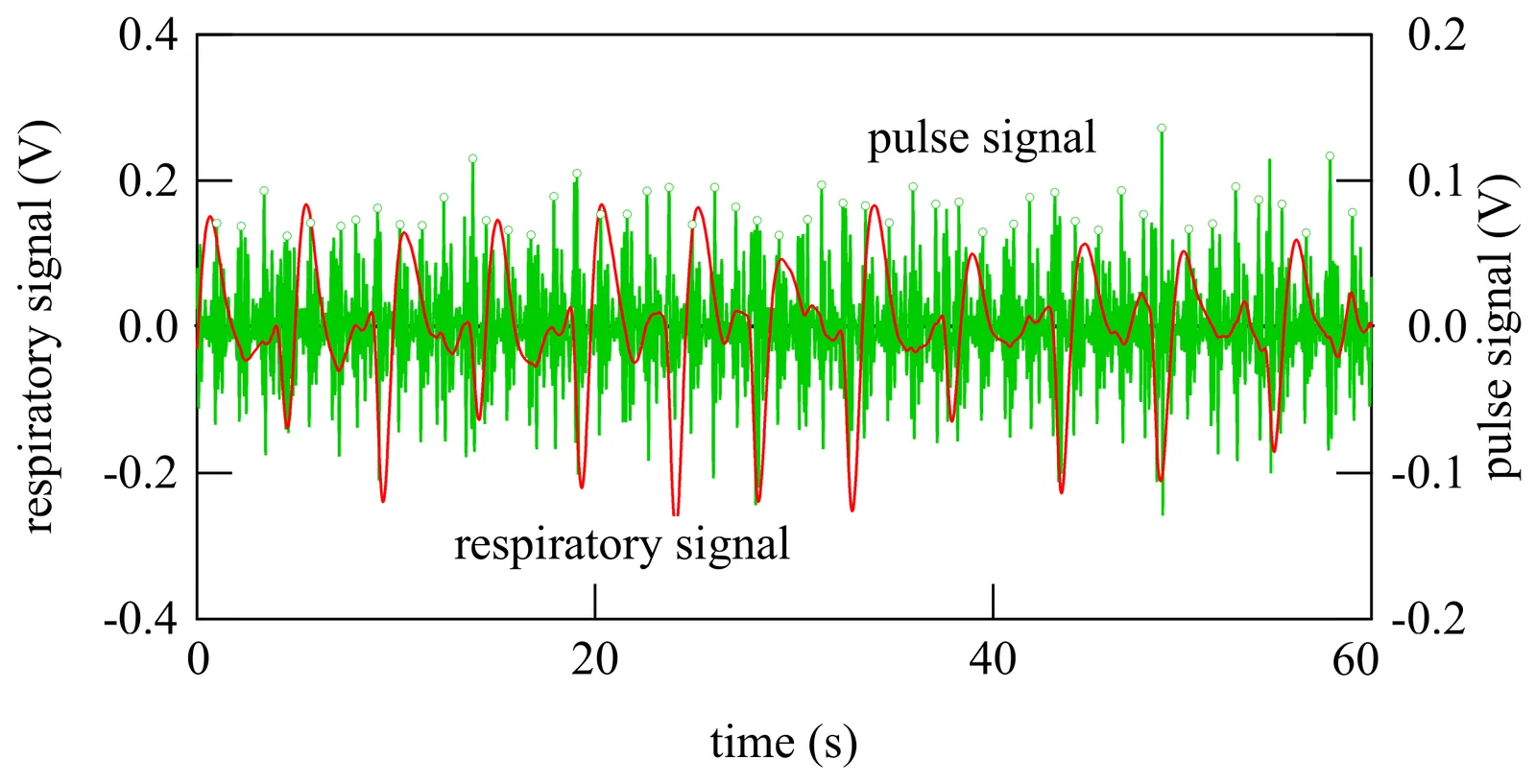
Sample 60-s of respiratory and pulse signals obtained from piezoelectric PLLA braided cord sensor. The red line represents the respiratory signal, and the green line represents the pulse signal.

Figure 8 shows a representative signal recorded from the piezoelectric PLLA braided cord sensor during an overnight clinical measurement. The sensor continuously recorded mechanical signals generated by the participant during sleep. In Figure 8, the detailed respiratory waveform obtained from the piezoelectric PLLA braided cord sensor is not clearly visible; therefore, a 60-s segment of the recorded signal was extracted, as shown in Figure 9. This enabled the respiratory waveform to be observed more clearly. Figure 10 shows a representative 60-s pulse signal extracted from the same sensor output. Figure 11 shows the respiration and pulse signals simultaneously obtained over a 60-s period. The two signal components were clearly distinguishable after the signal processing described in Section 3.3.

**Figure 12:**
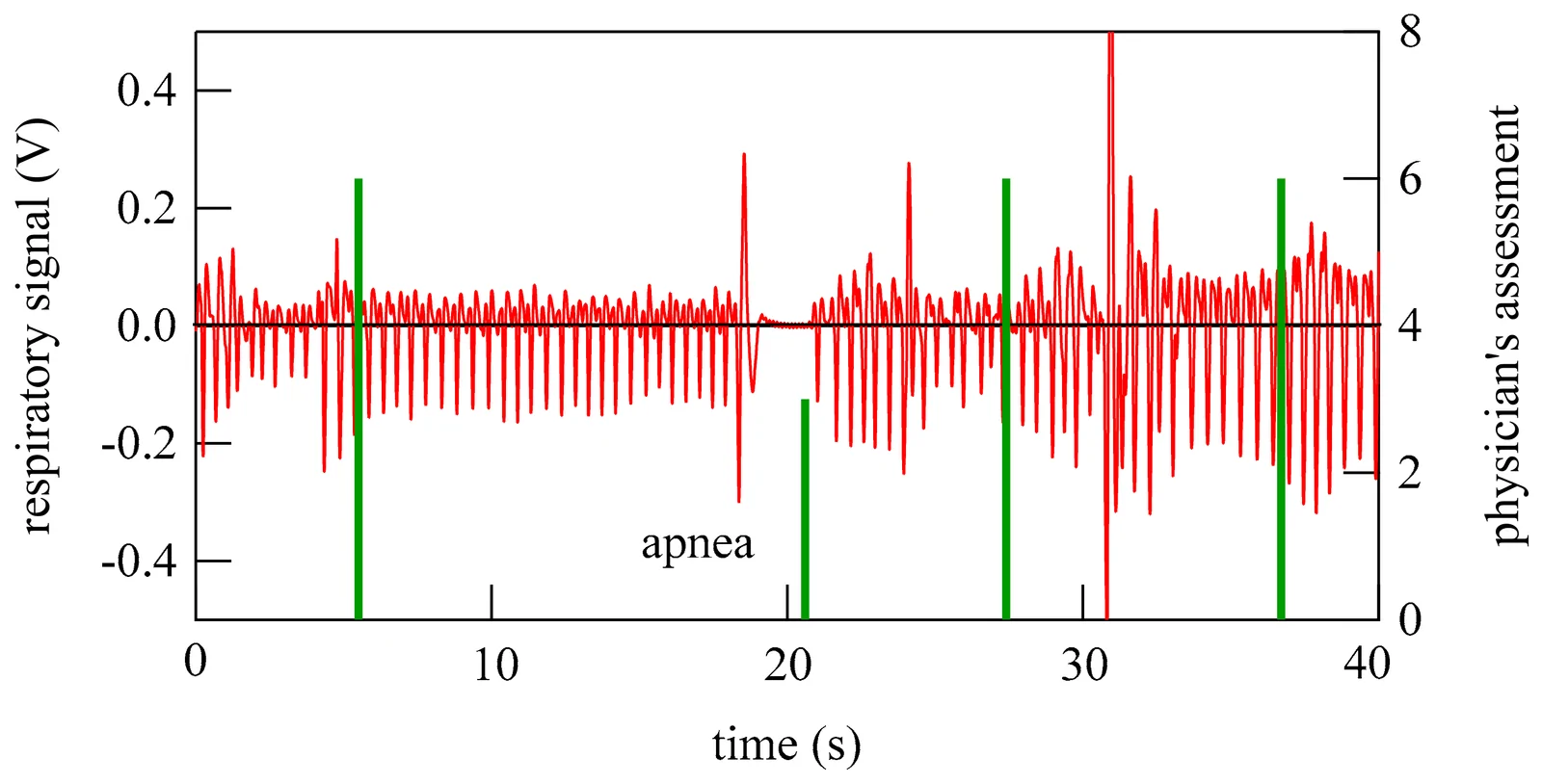
Comparison of the respiratory signal obtained from the piezoelectric PLLA braided cord sensor with the physician’s PSG-based assessment of apnea events. The red line represents the respiratory signal obtained from the piezoelectric PLLA braided cord sensor, and the green vertical lines indicate apnea events identified by the physician based on PSG.

**Figure 13:**
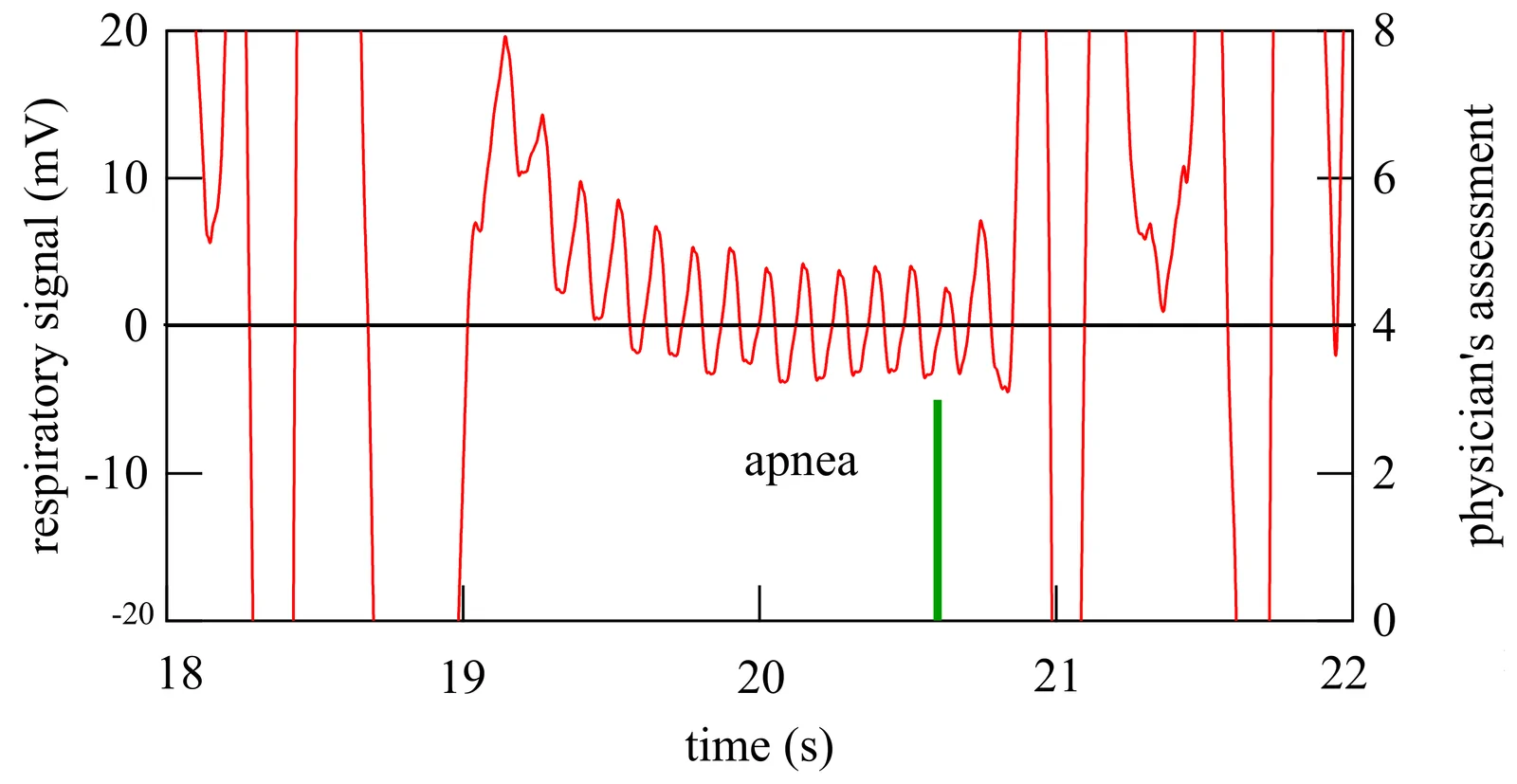
Enlarged view of the respiratory signal obtained from the piezoelectric PLLA braided cord sensor during an apnea event. The red line represents the respiratory signal obtained from the piezoelectric PLLA braided cord sensor, and the green vertical line indicates an apnea event identified by the physician based on PSG.

**Figure 14:**
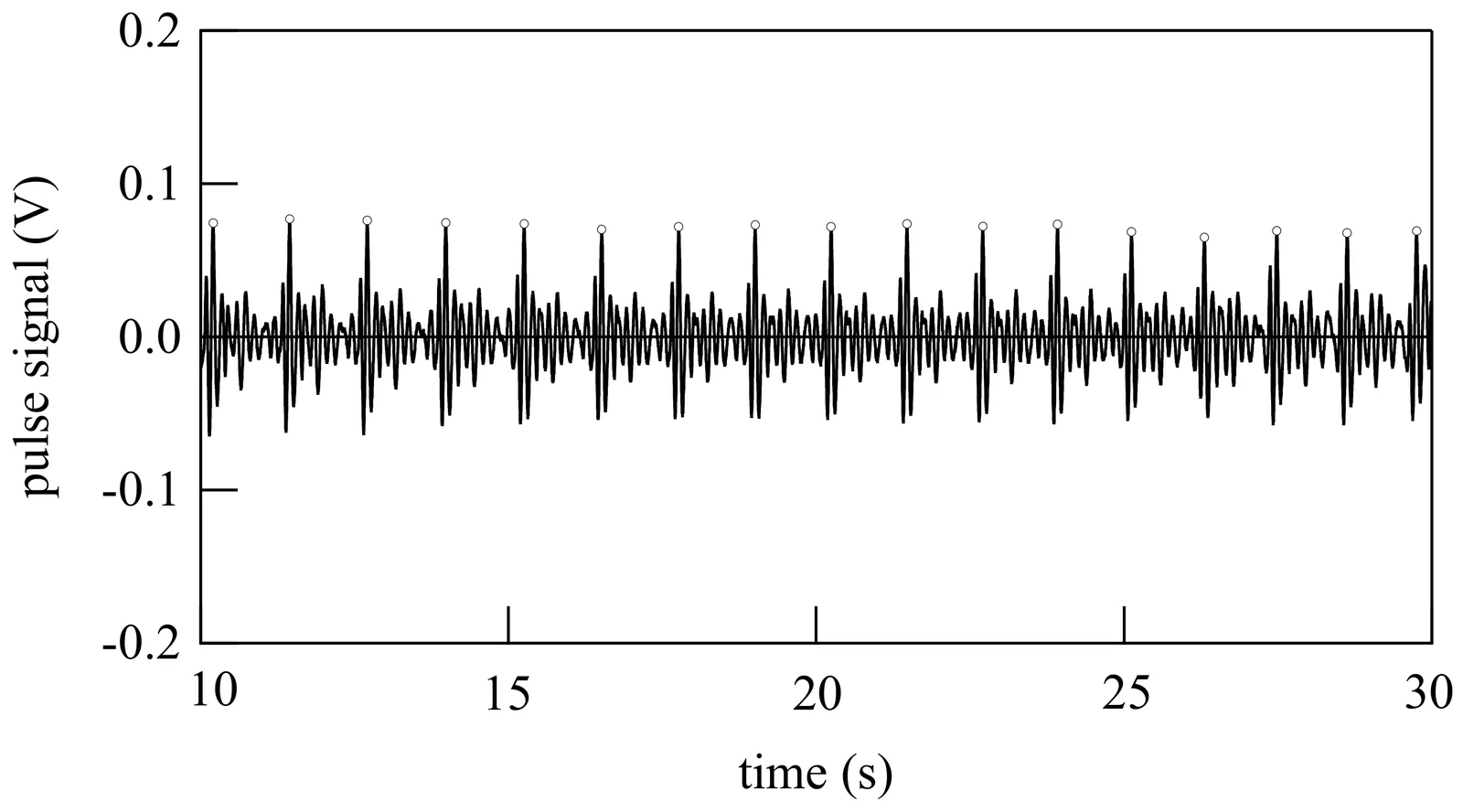
Pulse signal obtained from the piezoelectric PLLA braided cord sensor during the same apnea event shown in Figure 13.

**Figure 15:**
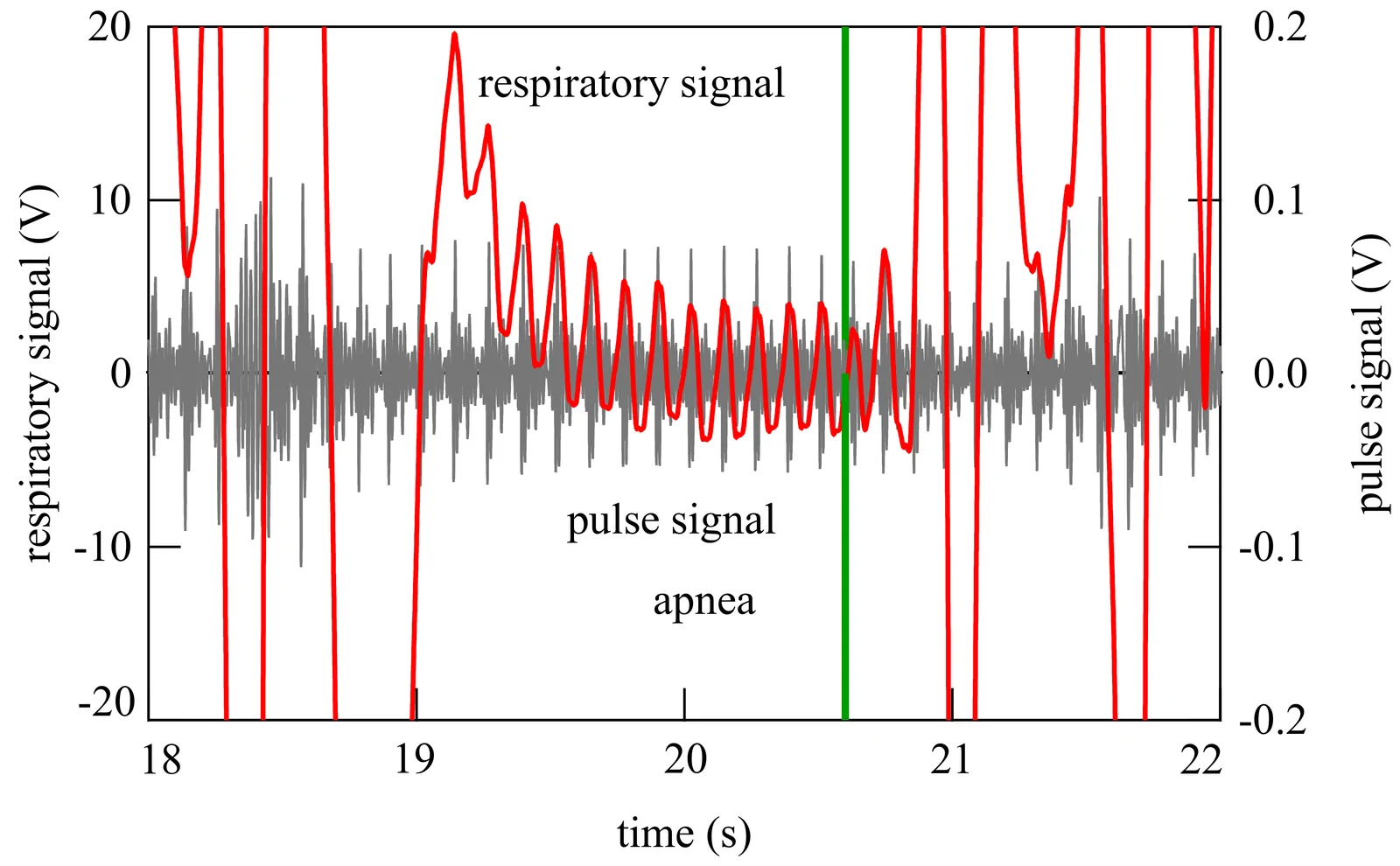
Superimposed respiratory and pulse signals recorded by the piezoelectric PLLA braided cord sensor during the apnea event shown in Figure 13. The respiratory signal amplitude decreases markedly during apnea, while a small periodic component with approximately the same frequency as the pulse signal remains detectable. The red line represents the respiratory signal, the gray line represents the pulse signal, and the green vertical line indicates an apnea event identified by the physician based on PSG.

Next, the respiratory signal obtained from the piezoelectric PLLA braided cord sensor was compared with the physician’s PSG-based assessment, as shown in Figure 12. The green bars represent the physician’s assessment based on PSG; a value of 6 represents normal breathing, whereas values below 6 represent apnea events. During the apnea event indicated by the central green bar, the amplitude of the respiratory signal decreased markedly. Figure 13 shows an enlarged view of the respiratory signal during this apnea event. Although the respiratory signal amplitude became very small, a small periodic component remained. Figure 14 shows the pulse signal recorded during the same period. No marked change in the amplitude of the pulse signal was observed. Figure 15 superimposes the respiratory and pulse signals during the apnea event. During the period in which the respiratory signal amplitude was markedly reduced, the remaining periodic component of the respiratory signal had approximately the same frequency as the pulse signal. These observations indicate that, during an apnea event, the respiratory signal amplitude decreases markedly while a pulse-related periodic component remains detectable. This characteristic was initially observed during the analysis of data from a small number of participants and subsequently used as the basis for developing the apnea detection algorithm.

### 4.2. Algorithm Development

**Figure 16:**
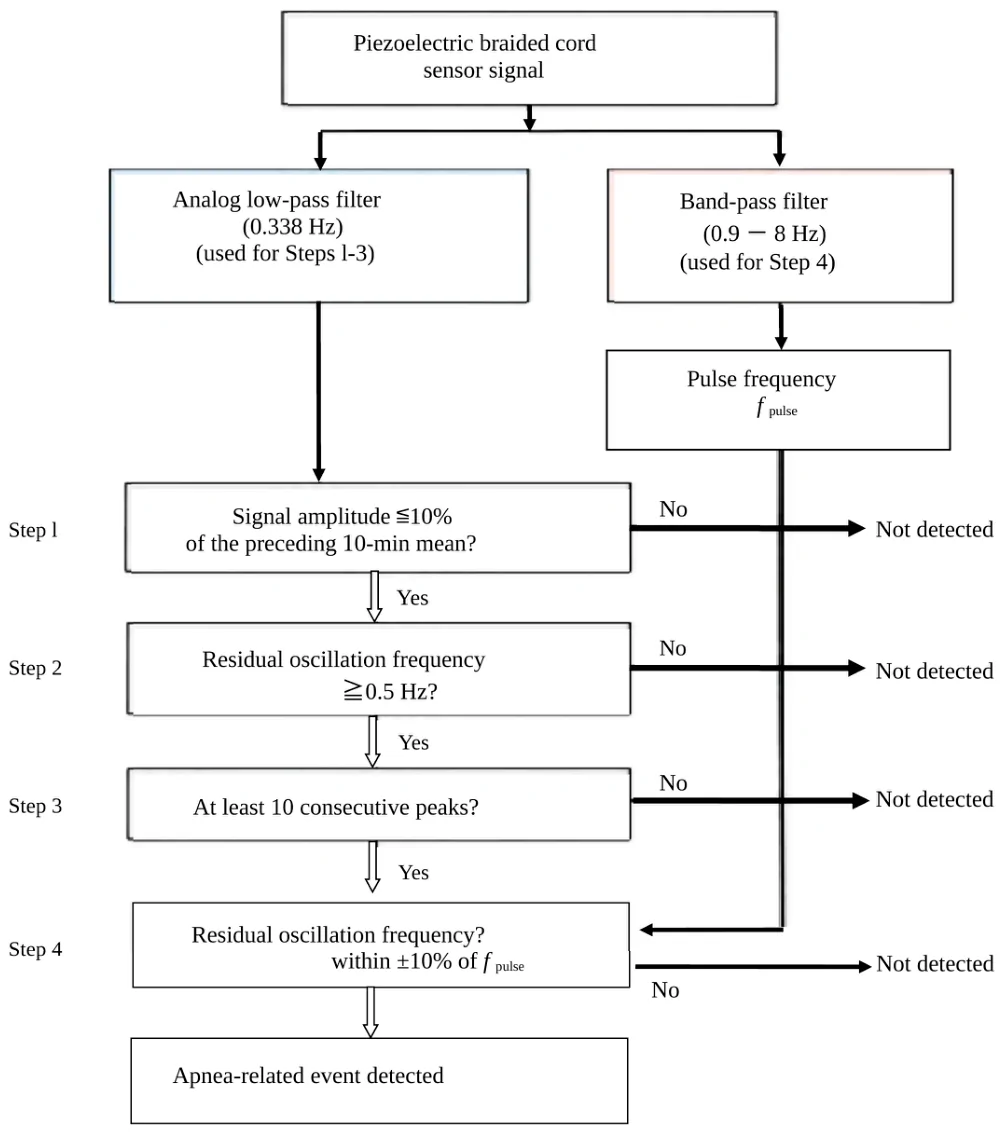
Flowchart of the proposed apnea detection algorithm showing the signal processing paths and the four detection criteria.

Based on the characteristic signal behavior observed in Figure 15, an algorithm was developed for apnea detection. The algorithm uses both the reduction in respiratory signal amplitude and the pulse-related periodic component remaining in the respiratory signal during apnea. The first three steps were applied to the output of the analog low-pass filter (0.338 Hz), whereas the pulse frequency used in the fourth step was independently determined from the 0.9–8 Hz band-pass-filtered signal. First, the amplitude of the respiratory signal was compared with the mean amplitude during the preceding 10 min. An apnea candidate was identified when the respiratory signal amplitude decreased to 10% or less of this mean value. The frequency of the respiratory signal was then calculated from the intervals between successive peaks. The event was considered an apnea candidate when the calculated frequency was 0.5 Hz or higher for at least 10 consecutive peaks. The frequency of the pulse signal was calculated independently from its peak intervals. Finally, an apnea event was identified when the frequency calculated from the respiratory signal agreed with the pulse frequency within ±10%. This criterion was used to identify the pulse-related periodic component remaining in the respiratory signal during apnea and was intended to reduce false-positive detections caused by changes in signal amplitude associated with body position or other movements. The algorithm was applied to the sensor data recorded during routine PSG examinations to detect apnea-related signal changes. The flowchart of the proposed apnea detection algorithm, including the signal processing paths and the four detection criteria, is shown in Figure 16.

### 4.3. Results

Table 3 compares the number of apnea events detected by the piezoelectric PLLA braided cord sensor using the algorithm described in Section 4.2 with the number identified by the physician based on PSG. The comparison was performed using event counts within consecutive 5-min intervals, rather than by matching the occurrence times of individual events. For each participant, the table lists the SAS severity classification determined by the physician, basic participant characteristics, the number of apnea events identified by the physician based on PSG, the number of sensor-detected events counted as corresponding to PSG events based on the 5-min interval comparison, and the number of additional events detected by the sensor. The agreement rate was calculated by summing the smaller of the sensor-detected and PSG-identified event counts within each 5-min interval and dividing this sum by the total number of sensor-detected events across all intervals. The comparison of event counts within 5-min intervals does not establish whether individual PSG-identified apnea events were detected by the proposed sensor, because event-by-event temporal matching was not performed. However, in some 5-min intervals, the number of apnea-like events detected by the sensor exceeded the number of apnea events identified by the physician based on PSG. Overall, the event-count agreement rates based on 5-min intervals were approximately 90% across the 18 evaluated participants. These results indicate good agreement in apnea event counts within 5-min intervals but do not demonstrate event-by-event correspondence between the proposed sensor and PSG. We were informed after completion of the measurements that the last five participants listed in Table 3 were healthy individuals. For these five participants, physician-based PSG apnea event assessments were not available, and they were therefore excluded from the agreement rate calculation. The apnea-like events detected by the proposed sensor in these participants could not be evaluated against physician-identified apnea events. For these participants, the proposed sensor detected five or fewer apnea-like events per night. The number of apnea-like events detected in these healthy participants was generally lower than that observed in the participants with SAS.

Overall, the comparison of apnea event counts within 5-min intervals showed good agreement between the braided cord sensor and physician-based PSG assessments for the 18 evaluated participants. Individual apnea events were not temporally matched between the two methods. These findings support the potential of the proposed sensor for unobtrusive monitoring of respiration, pulse, and apnea-related events during sleep.

## 5. Conclusions

In this study, we developed a bed-sheet sensor incorporating piezoelectric poly-L-lactic acid braided cords and investigated its potential application to sleep apnea monitoring. FEM analysis was used to obtain qualitative guidance for sensor development, and a multi-orientation stitching configuration was developed through iterative prototyping and experimental evaluation. The developed sensor enabled the detection of respiration- and pulse-related mechanical signals during sleep. We also observed that a pulse-related periodic component remained detectable when respiratory motion decreased during apnea. Based on this characteristic, an algorithm for detecting apnea-related signal changes was developed. Sensor signals were recorded from 23 participants during routine clinical PSG examinations. For 18 participants with available physician-based PSG apnea event assessments, sensor-detected apnea event counts were compared with PSG-based counts within 5-min intervals. The comparison showed good agreement in apnea event counts within 5-min intervals, although individual events were not temporally matched between the two methods.

These findings suggest that the proposed bed-sheet sensor may contribute to future monitoring of physiological signals during everyday sleep without requiring sensors to be attached to the body. Further investigations, including evaluation using independent data, are needed to assess its detection performance and explore its potential for long-term home-based monitoring.

## Figures and Tables

**Table 1:** Characteristics of the participants in the PSG measurements.

Subjects	Gender	Age	Height (cm)	Weight (kg)	BMI
1	male	34	163	96	36
2	female	81	159	58	23
3	male	39	173	67	22
4	male	15	164	49	18
5	male	47	181	68	21
6	male	46	170	90	31
7	male	58	170	64	22
8	female	38	170	82	28
9	male	61	167	69	25
10	male	64	175	98	32
11	female	58	163	66	25
12	male	51	170	90	31
13	female	54	171	68	23
14	male	73	174	82	27
15	male	40	173	68	23
16	male	29	180	70	22
17	male	50	181	80	24
18	male	26	172	76	26
19	female	49	160	50	20
20	male	35	155	60	25
21	male	25	170	52	18
22	male	35	155	60	25
23	male	59	171	74	25

**Table 2:** Classification of sleep apnea syndrome based on the apnea–hypopnea index (AHI).

Item	Definition/Criterion
AHI	(Number of apneas + hypopneas)/sleep time (h)
Apnea	≥10 s complete cessation of airflow
Hypopnea	≥10 s airflow reduction ≥30% with 3–4% SpO_2_ drop or arousal
Normal	AHI < 5
Mild	5 ≤ AHI < 15
Moderate	15 ≤ AHI < 30
Severe	AHI ≥ 30

**Table 3:** Comparison of apnea event counts obtained from the piezoelectric braided cord sensor and physician-based PSG assessments using 5-min intervals.

Subject	Severity Index	Gender	Age	Height (cm)	Weight (kg)	BMI	Number of Times of Apnea Event Judged by Physician	Corresponding Event Counts Based on 5-min Intervals	Number of Additional Events Detected by the Braided Cord Sensor	Event-Count Agreement Rate Based on 5-min Intervals (%)
1	Severe	male	34	163	96	36	218	218	20	92
2	Mild	female	81	159	58	23	67	67	10	87
3	Moderate	male	39	173	67	22	156	156	5	97
4	Mild	male	15	164	49	18	99	99	20	83
5	Moderate	male	47	181	68	21	113	113	7	94
6	Moderate	male	46	170	90	31	123	123	17	88
7	Moderate	male	58	170	64	22	99	99	2	98
8	Mild	female	38	170	82	28	84	84	11	88
9	Moderate	male	61	167	69	25	120	120	18	87
10	Severe	male	64	175	98	32	199	199	14	93
11	Severe	female	58	163	66	25	239	239	15	94
12	Severe	male	51	170	90	31	278	278	18	94
13	Severe	female	54	171	68	23	240	240	14	95
14	Severe	male	73	173.5	82	27	233	233	51	82
15	Moderate	male	40	173	68	23	129	129	15	90
16	Mild	male	29	180	70	22	79	79	12	87
17	Mild	male	50	181	80	24	69	69	5	93
18	Severe	male	26	172	76	26	189	189	10	95
19	Normal	female	49	160	50	20	Not assessed	N/A	3	N/A
20	Normal	male	35	155	60	25	Not assessed	N/A	5	N/A
21	Normal	male	25	170	52	18	Not assessed	N/A	5	N/A
22	Normal	male	35	155	60	25	Not assessed	N/A	3	N/A
23	Normal	male	59	171	74	25	Not assessed	N/A	2	N/A

Note: The agreement rate was calculated using data from the 18 participants for whom physician-based PSG apnea event assessments were available. The remaining five participants were excluded from this calculation. Agreement was evaluated using apnea event counts within 5-min intervals, without event-by-event temporal matching. For Subjects 19–23, the values in the “Number of additional events detected by the braided cord sensor” column represent the total numbers of apnea-like events detected by the sensor, rather than additional events relative to PSG assessments.

## Data Availability

The data presented in this study are available on request from the corresponding author. The data are not publicly available due to privacy and ethical restrictions related to the clinical study.
